# A Circular Bioeconomy Model for Oaxaca: Integrating Entomophagy and Zootechnical Validation in Small-Scale Tilapia Farming

**DOI:** 10.3390/insects17020225

**Published:** 2026-02-21

**Authors:** Tamara Aquino-Aguilar, Yolanda Donají Ortiz-Hernández, Marco Aurelio Acevedo-Ortiz, Teodulfo Aquino-Bolaños, Gema Lugo-Espinosa, Jesús Andrés Morales-López, Salatiel Velasco-Pérez

**Affiliations:** 1 Secretaría de Ciencia, Humanidades, Tecnología e Innovación (SECIHTI), Instituto Politécnico Nacional, CIIDIR Oaxaca, Santa Cruz Xoxocotlán 71230, Oaxaca, Mexico; taquinoa2000@alumno.ipn.mx (T.A.-A.); glugoe@ipn.mx (G.L.-E.); jmoralesl2100@alumno.ipn.mx (J.A.M.-L.); svelascop2500@alumno.ipn.mx (S.V.-P.); 2 Instituto Politécnico Nacional, CIIDIR Oaxaca, Santa Cruz Xoxocotlán 71230, Oaxaca, Mexico; taquino@ipn.mx

**Keywords:** operational sustainability, sustainable food systems, food sovereignty, edible insects, aquaculture feed

## Abstract

Global population growth necessitates sustainable food systems, positioning Circular Bioeconomy as a key transition framework. In Oaxaca, Mexico, semi-intensive tilapia aquaculture faces economic viability issues due to a critical reliance on expensive external commercial feeds. This study proposes a “Backyard Integrated System” specifically designed for rural contexts with limited capitalization, connecting traditional entomophagy with aquaculture to reduce operational costs and close nutrient cycles. Using a mixed-method approach, we first conducted a sociocultural diagnosis (*n* = 140), revealing a 97.14% acceptance of insect consumption. Subsequently, to validate technical viability, a long-term (280-day) feeding trial was conducted using standardized insect meals (*Tenebrio molitor* and *Acheta domesticus*) as total substitutes (100%) for commercial feed in Nile tilapia (*Oreochromis niloticus*) diets. Results showed a Feed Conversion Ratio (FCR) of 1.61–1.62, comparable to the commercial control (*p* > 0.05), while significantly enhancing fillet protein content. Crucially, microbiological analysis confirmed the absence of pathogens in the final product, empirically validating the safety of the waste-to-feed cycle. Consequently, this strategy ensures food sovereignty, decouples producers from volatile external markets, and offers a scalable solution for community resilience without compromising food safety.

## Simple Summary

In Oaxaca, Mexico, small-scale fish farmers face a major economic challenge: commercial feed is too expensive, accounting for most of their production costs. To solve this, we developed a “Backyard Integrated System” specifically designed for rural families with limited resources. This model connects the local tradition of eating insects with fish farming. Instead of buying expensive feed, farmers can use their own household organic waste to raise insects like mealworms and crickets, which are then turned into flour to feed Nile tilapia. Our study confirmed three key findings: first, the local population widely accepts insect consumption; second, fish fed entirely on insect flour grow just as well as those fed commercial brands and contain more protein; and third, the final fish meat is free of harmful bacteria, proving that this waste-to-feed cycle produces safe food. This system is valuable to society because it allows communities to produce high-quality protein at near-zero cost, recycling waste and breaking their dependence on volatile external markets.

## 1. Introduction

The projected increase in the global population, expected to reach 10 billion people by 2050 [1], poses a critical demand for food that could exceed 50% of current production [1,2]. Under these circumstances, pressure on finite environmental resources, the exacerbation of food insecurity, and ecological degradation impose an urgent need to redefine economic management models.

Given this scenario, Circular Bioeconomy has consolidated itself as the ideal framework to guide this transition [3,4]. This paradigm demands that agri-food systems abandon linear extractive schemes to adopt regenerative models, integrating efficiency in the use of renewable biological resources (Bioeconomy) with the principles of regeneration and closing cycles inherent to the Circular Economy [3,4].

Under this logic of minimizing the carbon footprint and integral valorization of by-products, diversifying the protein matrix becomes an absolute necessity [5,6,7]. Consequently, insect utilization emerges as a strategic solution for dual purposes (direct human consumption and essential animal nutrition). This alternative aligns with Circular Bioeconomy as operational sustainability: not only does it require considerably fewer water and land resources, but its carbon footprint is also notably lower (14 kg CO_2_-eq) compared to conventional livestock (88 kg CO_2_-eq) [8,9]. Its systematic relevance lies in its high bioconversion capacity, allowing the transformation of organic waste into biomass of high nutritional value, thus operationalizing the closing of nutrient cycles.

This theoretical potential is substantiated by experimental evidence supporting the valorization of organic waste, agricultural by-products, and agro-food residues as feed for edible insects [10]. Specifically, *Tenebrio molitor* creates a functional link in this cycle, as it can develop effectively on diets based on agro-industrial by-products—such as distillery grains, brewer’s yeast, bread waste, and cereal residues—without affecting survival or growth rates [11,12]. Furthermore, this species is capable of bioconverting lignocellulosic materials and horticultural remains into high-biological-value protein [13]. Likewise, for *Acheta domesticus*, it has been reported that diets formulated with mixed food waste and agricultural by-products rich in carbohydrates and proteins result in adequate growth rates, survival, and feed conversion efficiency [14].

The application of these Circular Bioeconomy principles finds an immediate technological response in aquaculture, a sector projecting global growth of 17.4% by 2032 [15,16]. To ensure its sustainability, promoting water recycling and nutrient recovery systems is essential. In this sense, aquaponics—integrating fish production with plant cultivation—emerges as the ideal technological basis for this transition.

Specifically in the case of Mexico, tilapia (*Oreochromis niloticus*) farming represents the second most important fish activity [17,18,19], being fundamental for the regional economy. However, it faces severe limitations: predominant cultivation systems (semi-intensive) [17] rely crucially on external inputs, where commercial feed constitutes the main limiting factor, representing between 50% and 70% of operational costs [17,20]. This dependence on the global market, often sustained by the overexploitation of fishmeal [20], compromises the financial viability of farms [17], a situation aggravated by water scarcity and high energy costs that hinder technification [17]. Facing this fragility, integrating the entomophagic tradition into animal nutrition arises as a necessary strategy to revalorize local resources.

The viability of implementing insect-based Circular Bioeconomy solutions is limited to territories with deep-rooted cultural acceptance [21,22,23,24,25,26,27,28,29,30,31,32]. Oaxaca, Mexico, offers a unique biocultural context for this purpose: it harbors a vast biodiversity of edible arthropods (between 500 and 600 species) [27,33] distributed across its eight regions. While entomophagic practice is transversal in the state, species such as the “chinche de monte” (*Edessa* spp.) in the Mixteca region, or “chapulines” (*Sphenarium* spp.), “chicatanas” (*Atta mexicana*), and “gusanos de maguey” (*Comadia redtenbacheri*) in the Central Valleys, exemplify a functional diversity that mitigates the barrier of neophobia [22,28,29,30,31]. They possess high nutritional and biological value that actively contributes to local food sovereignty, being consistently valued by both the Oaxacan population and tourist circuits [24,26,34,35].

However, reliance on wild harvesting imposes a crucial limitation on the productive potential of this ancestral resource [30]. The acquisition of these insects is eminently seasonal, preventing a constant supply of high-value protein and carrying significant risks of overexploitation of native populations [29,36]. For insects to transition from being a seasonal ancestral resource to a pillar of modern food security, an integral validation articulating biocultural knowledge with zootechnical viability is required. Although flour substitution has been technically explored, there is a gap in the literature on how to integrate these findings into a viable circular economy model for rural communities [3,15].

This research addresses this gap by proposing the Backyard Integrated System Model, conceptualized as a system integrating aquaculture with controlled insect rearing. Crucially, this model is designed for rural contexts characterized by limited capitalization, operating under a logic of “self-generation” where low-impact organic waste is revalorized to substitute expensive commercial inputs. To support the technical viability of this proposal, the research builds upon the nutritional validation of native flours previously conducted by our group [8,28,37] and experimental protein substitution data [8,17]. Therefore, the objective is to demonstrate how the articulation of biocultural practice with zootechnical validation constitutes the missing link to guarantee a source of local, safe, and high-value protein, mitigating the economic dependency of Oaxacan aquaculture farms on high-cost external feed inputs, and strengthening food sovereignty by incorporating endemic resources into autonomous and culturally relevant production systems.

## 2. Materials and Methods

**Figure 1:**
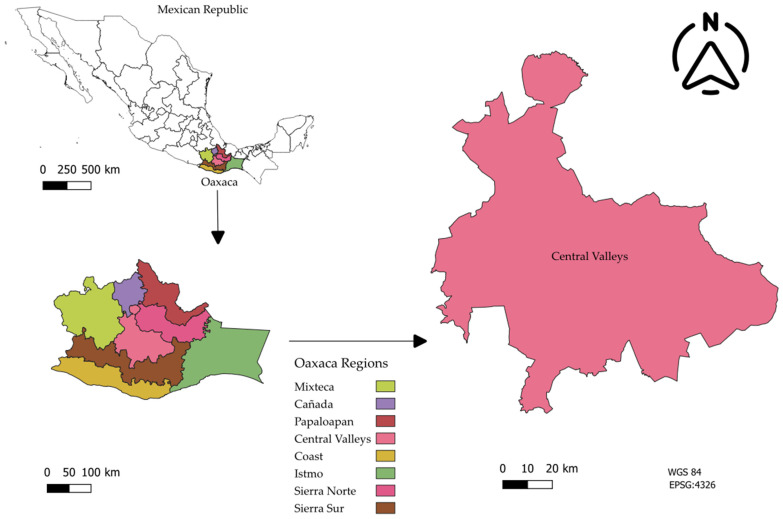
Geographic location of the Central Valleys of Oaxaca, Mexico.

The research was conducted under a mixed-methods design [38] in the Central Valleys region of Oaxaca (Figure 1). Located in the central part of the state, this strategic territory spans 9480 km^2^ and is situated within the following coordinates: Latitude: 17°18′00″ N–16°36′00″ N and Longitude: 96°57′00″ W–96°17′00″ W. The region comprise 121 municipalities, 26 of which are considered key municipalities for the state’s economic dynamics [39]. The methodology articulated three complementary phases: sociocultural diagnosis, nutritional validation of inputs, and experimental zootechnical evaluation.

### 2.1. Sociocultural Diagnosis

**Figure A1:**
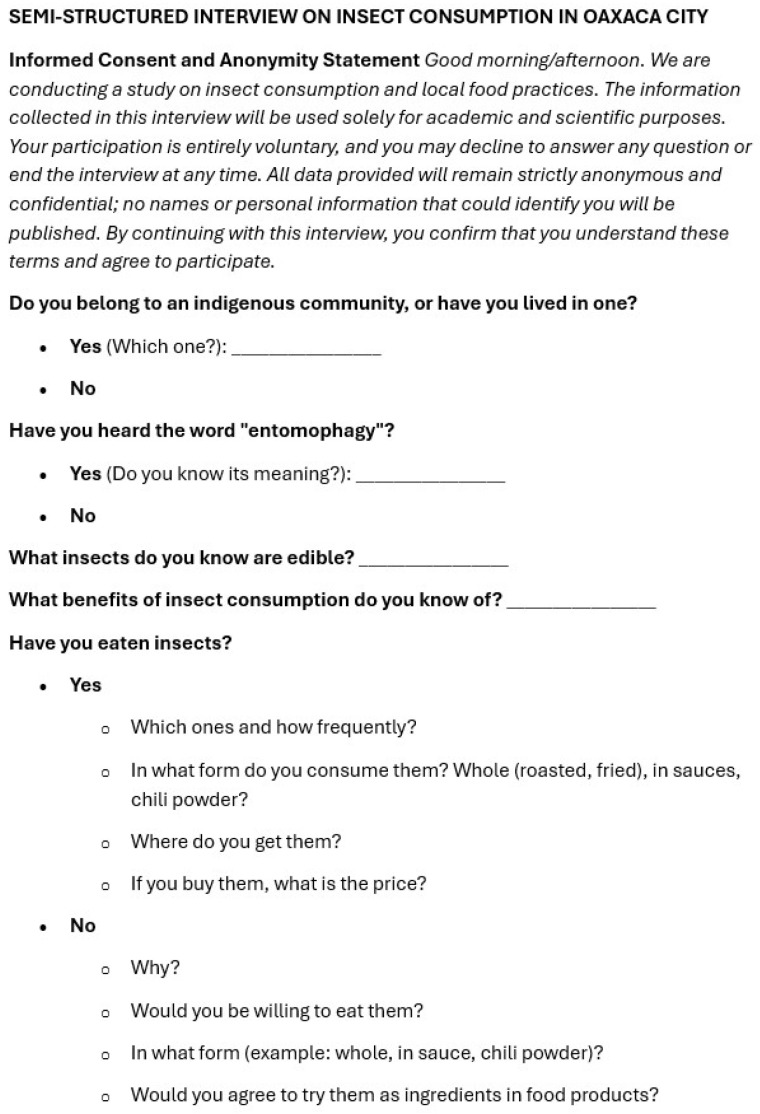
Informed consent and semi-structured interview.

Primary information collection was carried out during January 2025 using a non-probabilistic sample of 140 informants in representative markets of the metropolitan area. The study population was stratified into three key groups: vendors (insects and traditional food), local diners, and tourists. The procedure was structured in two stages: (a) General Survey, based on semi-structured interviews (Figure A1) to determine consumption prevalence, preferred species, and acquisition modes; and (b) Focused Study, directed at key informants through participant observation [22,29,30,31,40].

To define the economic viability of protein substitution, a situational analysis of local aquaculture [17] was integrated. This baseline identified that semi-intensive systems in the region face critical profitability limitations derived from high feed and energy costs, thus underscoring the urgency of the proposed bioeconomic model [17].

### 2.2. Nutritional and Economical Validation of Inputs

The selection and processing of insects utilized in the experimental diets were based on validation protocols reported in previous works [8,37]. Flours from native species (*Sphenarium* spp., *A. mexicana*, *Q. gigas*, and *Edessa* spp.) collected in Oaxaca were used. To guarantee standardization, a cleaning process with distilled water was followed, along with sacrifice by thermal shock (4 °C/30 min and 70 °C/3 min), drying in an oven (Ecoshel 9025H, Denver, CO, USA), at 65 °C for 24 h, and grinding at 10,000 rpm.

It is important to specify that, for the specific case of the “chinche de campo” (*Edessa* spp.), nutritional values are based on the bromatological characterization previously performed by this working group in the Mixteca region [28]. Consequently, a new collection of this taxon was not carried out in the Central Valleys zone; instead, the protein quality and functional parameters already consolidated in said research were extrapolated to provide certainty to the model’s input values.

Biological Conversion and Economic Assessment: This processing stage was critical for defining the economic viability of the model. A specific analysis of the biological conversion factor was conducted, recognizing that insects possess high moisture content requiring significant fresh biomass to yield dry flour. Based on small-scale rearing metrics, specific drying yields were determined (32% for *T. molitor* and 27% for *A. domesticus*). Consequently, 3.12 kg and 3.70 kg of fresh biomass, respectively, are required to produce a single kilogram of shelf-stable flour. These conversion rates were used to estimate the production costs per kg of dry flour intended for aquaculture feed, contrasting them with the market price of commercial feed (Table 1).

Nutritional Characterization and Safety: For diet formulation, no new chemical characterizations were performed; instead, previously certified quality profiles were utilized [28,37]. Crude protein values were based on the Kjeldahl method (NMX-F-608-NORMEX-2011) [41,42], using validated conversion factors of 5.60 for orthopterans/cicadas and 4.76 for hemipterans.

Regarding safety, the decision to use processed flours instead of fresh or whole insects was based on previous microbiological evaluation [8,28,37], which demonstrated that the thermal processing of flours guarantees their safety. This processing is crucial as it ensures compliance with the limits of Mexican standards NOM-092-SSA1-1994 (mesophiles) [43], NOM-113-SSA1-1994 (coliforms) [44] and NOM-210-SSA1-2014 (fungi and yeasts) [45]. Techno-functional properties for pelletizing (Hausner and Carr indices, water/oil retention) were equally supported by previous reports [28,37,46].

### 2.3. Experimental Zootechnical Evaluation

To evaluate the efficiency of insect flours as a sustainable substitute, a long-term (280-day) Nile tilapia (*O. niloticus*) fattening experiment was designed in a Recirculating Aquaculture System (RAS) [47] under greenhouse conditions during 2024.

Experimental Conditions: The system consisted of 18 fiberglass tanks (730 L) with biological and mechanical filtration. Water quality parameters (temperature, pH, dissolved oxygen) were monitored daily to ensure optimal conditions [48]. Water temperature was maintained between 23.27 ± 2.16 °C and 29.01 ± 2.16 °C; pH between 7.3 ± 1.6 and 8.4 ± 1.6; DO between 4.5 and 6.5 mg L^−1^; hardness < 75 mg L^−1^; carbonates < 120 mg L^−1^; alkalinity < 80 mg L^−1^; nitrites < 0.003 mg L^−1^; and nitrates < 8.005 mg L^−1^. Fecal matter from each tank was siphoned every five days, and 50% water exchanges were performed every 20 days.

Diets and Management: Fingerlings with an initial weight of 2.13 g were used, distributed at a density of 12 fish per tank with three replicates per treatment [48]. Three experimental diets were evaluated: D1 (100% *Tenebrio molitor*) and D2 (100% *Acheta domesticus*) alongside the commercial control D3 (Nutripec^®^) (Table 2). All diets were processed via pelletizing and drying at 60 °C. Feeding was supplied three times a day at 5% of body biomass, with adjustments every 20 days [47].

Growth Parameters: Biometric measurements were taken every twenty days to calculate zootechnical indices: Weight Gain (Wg) = final weight − initial weight; Feed Conversion Ratio (FCR) = feed apparently consumed (g)/weight gained (g); Specific Growth Rate (SGR) = ((ln final weight − ln initial weight)/time) × 100; and Condition Factor (K) = (final weight/final length^3^) × 100 [45].

Microbiological Quality Evaluation: To determine the microbiological quality of each diet treatment on the final product, analysis of the fish fillet was performed following NOM-210-SSA1-2014 [45]. A 100 g fish fillet sample was ground in a BLST4655-013 blender (OSTER^®^, Milwaukee, WI, USA), and homogenized via agitation. From the homogenized sample, a 10 g aliquot was diluted in 90 mL of 1% peptone water. Serial dilutions were prepared using 9 mL of sterile peptone water and 1 mL of the stock solution. From the final dilution, a 1 mL aliquot was plated on Plate Count Agar (PCA) and Potato Dextrose Agar (PDA) both from MCD Lab^®^, Oaxaca, Mexico, and Universal Chromogenic Agar (UCA) (TM MEDIA^®^, Delhi, India). PCA and UCA plates were incubated at 37 °C for 48 h, and PDA plates at 28 °C for 72 h. Results were expressed as CFU/g.

Proximal Analysis: To determine the effect of the experimental diets on the protein, fat, carbohydrate, and moisture content, a proximal analysis of the *O. niloticus* fillet was performed in triplicate, in accordance with the Official Mexican Standard NOM-051-SCFI/SSA1-2010 [49]. For this experiment, 200 g of fillet were utilized. The specific methodologies employed included: the Kjeldahl method for protein quantification, the Mojonnier method for fat quantification, and the phenol-sulfuric acid method for carbohydrate determination. Moisture content was measured by drying samples in an oven for 24 h at 105 °C [49,50].

Statistical Analysis: Quantitative data obtained from proximal analyses and growth parameters were analyzed using Statistical Analysis Software (SAS^®^, v9.1, Cary, NC, USA). Analysis of Variance (ANOVA) was applied, and means were compared using Tukey’s test, considering a significance level of *p* < 0.05.

### 2.4. Bioeconomic Integration Model Design

Sociocultural, nutritional, and zootechnical findings were integrated under the Circular Bioeconomy framework, utilizing a data triangulation approach [38,51] to design the “Backyard Integrated System”, establishing matter and energy flows between the domestic subsystem (waste), the bioconversion module (insects), and the productive module (aquaculture). The conceptual design was validated by contrasting the technical requirements of the species (*O. niloticus*) with the seasonal availability and cultural acceptance documented in the previous phases.

## 3. Results and Discussion

### 3.1. Gastronomic Heritage and Biocultural Dynamics of Consumption

#### 3.1.1. Heritage Integration in the Value Chain

In the rural context of Oaxaca, traditional cuisine operates as an economic articulation mechanism between producing communities and urban consumption centers [22,52]. The research evidence that gastronomic tourism acts as a catalyst for the commercialization of insects in the metropolitan area, establishing a value chain where resources harvested in the periphery are integrated into the city’s culinary offer. This process has transformed insect consumption: from a subsistence strategy, it has evolved into an element of cultural prestige and tourist attraction [53,54].

While UNESCO establishes specific criteria for cultural heritage [55], insect consumption in this region, through symbolic interactionism that strengthens the social fabric [56], constitutes a current sociocultural practice implicitly assumed as identity heritage [57].

This is reflected in the in situ characterization of sales points, where significant physical integration is found in local markets. Insect stalls are frequently located adjacent to conventional meat sections (beef, pork, chicken) and traditional foods. This commercial arrangement is not coincidental; it denotes an implicit cultural classification where the insect is socially recognized as a primary protein source, comparable to conventional livestock, and not merely as a condiment or exotic curiosity.

**Figure 2:**
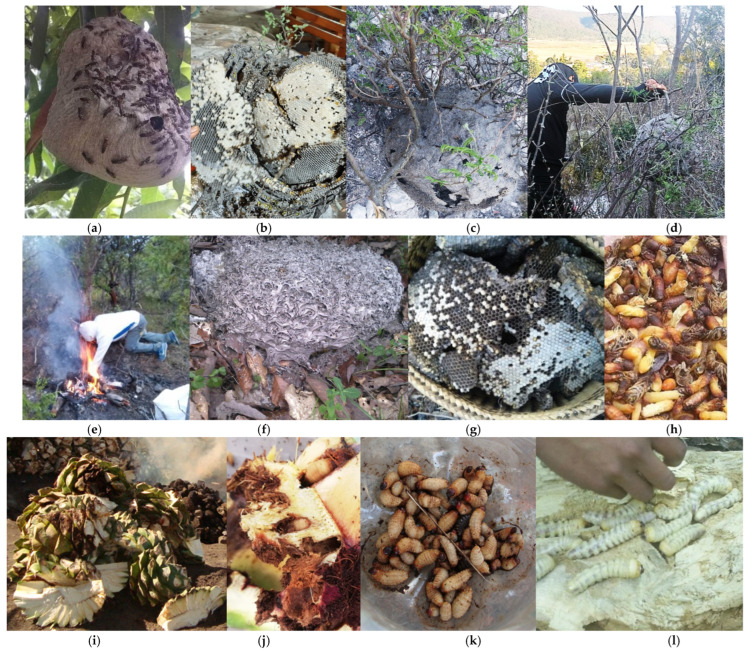
Traditional harvesting methods in Oaxaca. The extraction of hymenopterans is observed in arboreal (**a**–**d**) and subterranean (**e**–**h**) strata, as well as the collection of *Scyphophorus acupunctatus* associated with Agave (**i**–**k**) and *Mallodon chevrolatii* in decaying wood (**l**).

However, the supply chain remains fundamentally dependent on rural gatherers from Oaxaca’s different regions. This labor entails significant technical complexity that underscores reliance on natural cycles, ranging from the extraction of arboreal and subterranean wasp nests (*Vespidae*) to the manual harvest of *Scyphophorus acupunctatus* in infested agaves, or the extraction of *Mallodon chevrolatii* and *Aplagiognathus spinosus* larvae from decaying logs (Figure 2).

Once collected, the product is distributed both in marketplaces and through direct door-to-door sales. This dynamic responds to a dual demand: satisfying the local consumer who values historical authenticity [52,58], and attending to the tourist segment [22,59] seeking the gastronomic experience. In response, the culinary offer has diversified, integrating insects as high-value-added inputs in a wide range of products [30,53,60] (Table 3).

**Figure 3:**
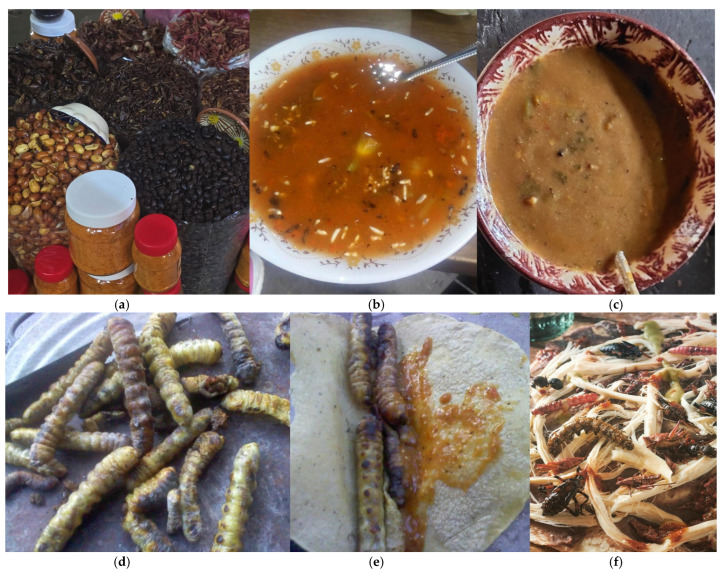
Culinary and commercial integration. From bulk sales of seasonal species such as *Sphenarium* spp., *Atta mexicana*, and *Comadia redtenbacheri* (**a**); through wasps in complex dishes like *chileatole* (**b**,**c**); *Mallodon chevrolatii* tacos on the griddle (*comal*) (**d**,**e**); to *Sphenarium* spp., *Atta mexicana*, and *Comadia redtenbacheri* in *tlayudas* (**d**–**f**), demonstrating their role as a central ingredient.

This versatility transcends simple bulk sales; insects assume a leading role by being integrated into complex dishes, demonstrating their status as a central ingredient rather than merely a peripheral garnish (Figure 3).

**Figure 4:**
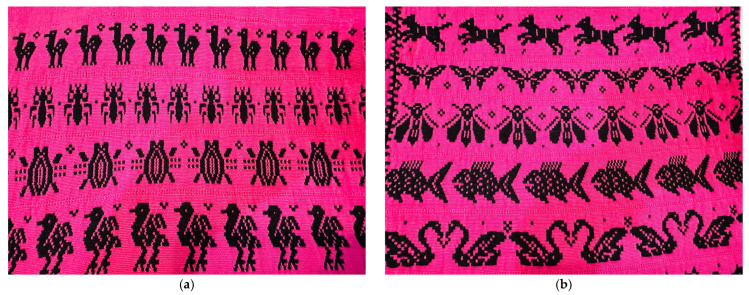
Textile iconography of insects. (**a**) Edible ants and bugs; (**b**) edible wasps.

On the other hand, the relevance of insects in the Oaxacan worldview is not limited to the food sphere; it also manifests in textile iconography. Artisans from rural communities capture the surrounding biodiversity in their textiles, integrating stylized representations of local entomological flora and fauna (Figure 4).

This graphic presence in traditional clothing functions as a cultural bioindicator: the community “weaves what it values” [61], evidencing that insects possess an ontological status extending beyond a simple caloric resource; they are an integral part of the collective identity and territory. This deep-rooted connection is a determining factor facilitating social acceptance of insect-based productive proposals.

#### 3.1.2. Cultural Roots and Prevalence of Entomophagy

Quantitative analysis confirmed the persistence and high acceptance of entomophagy in Oaxaca as a deeply rooted nutritional strategy [22]. 97.14% of interviewees (*n* = 140) in the metropolitan area reported having consumed insects [53,62]. Although consumption prevalence tends to be sporadic (41.80% consume rarely), absolute rejection is marginal (2.86%). A crucial finding for the viability of the proposed model is that 50% of consumers expressed willingness to ingest processed foods based on insect flour (such as enriched tilapia fillets), which mitigates the neophobia factor [6] and validates the industrial transformation pathway [7,31,40] (Table 4).

**Figure 5:**
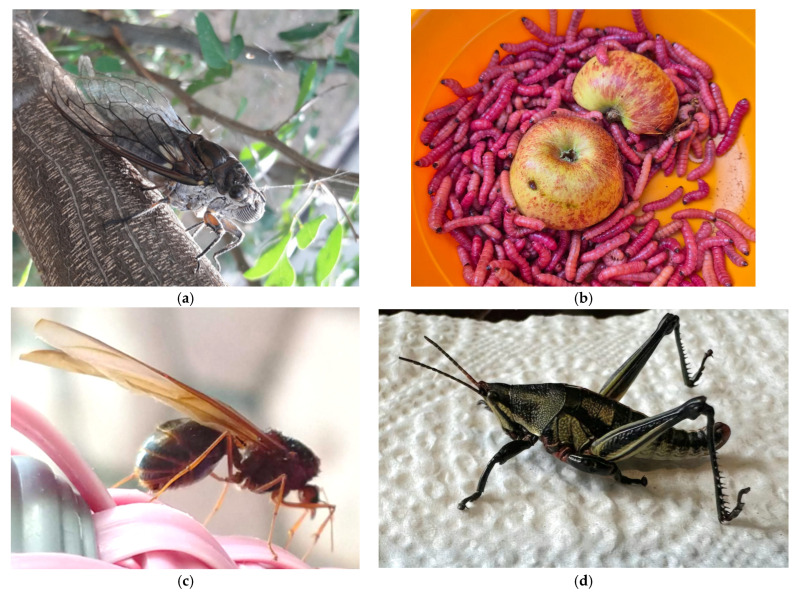
Edible insects of Oaxaca. (**a**) *Quesada gigas*; (**b**) *Comadia redtenbacheri*; (**c**) *Atta mexicana*; and (**d**) *Sphenarium* spp.

Barriers to consumption are primarily sociocultural (lack of knowledge, availability, lack of teaching at home, or perceived dislike of taste) rather than nutritional factors. The species with the highest market penetration are the “chapulin” (*Sphenarium* spp.), “chicatana” (*A. mexicana*), and “gusano de maguey” (*C. redtenbacheri*) in the Central Valleys, and the “chinche de campo” (*Edessa* spp.) in the Mixteca region (Figure 5 and Table 4) [36]. These species not only contribute to local food sovereignty but also possess high symbolic value associated with festivities and regional traditions.

#### 3.1.3. Seasonality: The Challenge for Food Sovereignty

Despite high demand and cultural value, the current system based on wild harvesting presents a critical structural limitation for the development of a Circular Bioeconomy: seasonality (Table 5). High-value species like the “Chicatana” (*A. mexicana*) are restricted to very short temporal windows (June-July), disproportionately elevating prices (up to $2500.00 MXN per kg) and generating pressure on natural populations.

**Figure 6:**
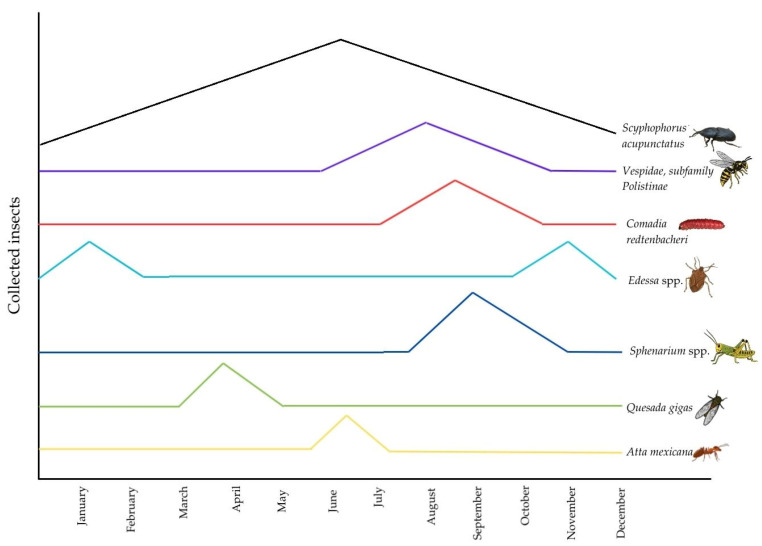
Seasonal availability pattern of edible insects collected in Oaxaca for potential aquaculture use.

While specific population density studies for these species in the region are scarce, scientific evidence regarding their seasonal availability patterns and geographic distribution is well-documented [30]. The natural availability of these insects follows a staggered succession pattern rather than a constant supply (Figure 6). While *Scyphophorus acupunctatus* exhibits a unique year-round presence due to its association with managed agave plantations, the majority of high-biomass species are strictly seasonal. The biological cycle begins with *Q. gigas* in spring (March–May), followed by the brief but intense emergence of *A. mexicana* (June–July). Subsequently, the peak biomass availability occurs during the rainy season and autumn (August–November), driven by *Sphenarium* spp., *C. redtenbacheri*, and Vespidae, concluding with *Edessa* spp. which covers the winter window (November–February).

Despite this apparent continuity, significant supply gaps remain. As shown in the graph, reliance on this succession implies constant logistical shifts between species, habitats, and regions, preventing the standardization required for a formal food industry. This extractive dependency entails risks of ecosystem overexploitation; therefore, the urgency of transitioning from a wild collection model towards controlled and sustainable zootechnical breeding practices to stabilize supply is evident.

### 3.2. Potential for Domestication of Native Species

To address the supply gaps identified in the seasonal analysis, transitioning to controlled rearing is imperative. However, unlike standardized commercial species, native taxa such as *Sphenarium* spp., *Comadia* spp., and *S. acupunctatus* require specific biotechnological adaptations to move from natural life cycles to production systems.

Biological characterization of *Sphenarium* spp., including nymphal stages, mating behavior, and oviposition, has been documented, providing a baseline for designing controlled reproduction protocols [63]. Regarding *C. redtenbacheri*, although pupal development and emergence have been achieved in laboratory settings, sustainable and scalable production for animal feed remains limited due to its long life cycle [64]. Conversely, *S. acupunctatus*, commonly known as the “agave weevil” and primarily studied as a pest, exhibits a complete cycle of 105–137 days. Its oviposition in agave tissues and extended larval development suggest the need for specialized rearing strategies to optimize biomass production [65].

Transitioning to controlled systems generally necessitates formulated artificial diets, substrate management, and strict control of environmental variables (temperature and humidity), which are essential for ensuring high survival and growth rates [66]. For long-cycle species like *Comadia* spp. and *S. acupunctatus*, mitigating predation and competition is key to efficiency. A critical strategy for low-cost production is the utilization of local agricultural by-products as feed and substrate. This approach reduces external input costs and promotes integrated circular systems, as demonstrated in mass rearing practices where agro-industrial residues are successfully used as basal diets [67].

While establishing these mass-rearing protocols for native biodiversity is a medium-term goal to ensure environmental sustainability, the immediate justification for their inclusion in the Bioeconomy model lies in their nutritional quality. Regardless of whether they are wild-harvested (current state) or farmed (future state), it is essential to first validate that their bromatological profile is superior to commercial inputs.

### 3.3. Analytical Validation of Raw Material: Nutrition and Safety

However, the viability of this productive transition depends not only on availability but on the intrinsic quality of the resource. Bromatological analyses confirmed that the cultural value of insects possesses solid scientific backing in terms of nutritional density [8,37,68].

**Figure 7:**
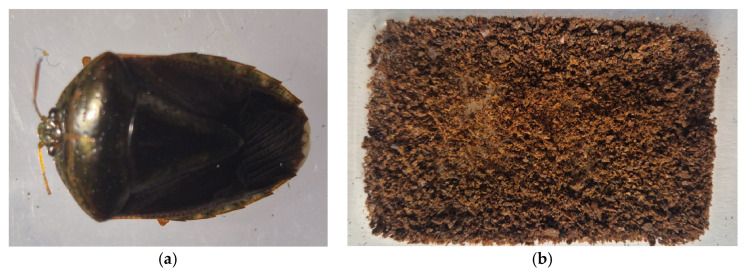
“Chinche de monte” (*Edessa* spp.) (**a**) Whole insect; (**b**) Processed flour.

Superior Nutritional Profile: Proximal characterization of processed native flours revealed competitive protein quality compared to conventional inputs [8,28,37]. “Chicharra” flour (*Q. gigas*, HQ) presented the highest protein content, reaching 36.00% [37]. This value, along with the 30.03% protein reported for the “chinche de monte” (*Edessa* spp.) (Figure 7), exceeds average values of conventional meats such as beef (18.4%), fish (18.3%), and chicken (22%) [28,69]. These findings validate that insect biomass constitutes a dense protein matrix capable of sustaining zootechnical requirements.

Safety Guarantee: A determining result for the model’s safety was the effectiveness of thermal processing. Although specimens in their raw state (*Edessa* spp.) presented detectable microbial loads, microbiological data demonstrated that transformation into flour (HS, HA, HQ) [37] eliminates 100% of coliforms, molds, and yeasts. This confirms that simple processing is sufficient to comply with sanitary regulations and overcome safety barriers in feed production [28,37].

**Figure 8:**
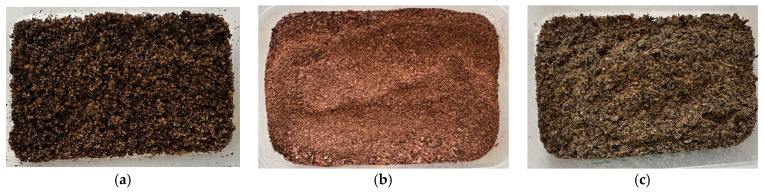
Processed flours of (**a**) *Atta mexicana* (HA), (**b**) *Sphenarium* spp. (HS) and (**c**) *Quesada gigas* (HQ).

Additionally, the techno-functional properties of the flours, such as the high emulsifying capacity of *Q. gigas* (HQ) (93.47%) and water/oil retention indices in *Sphenarium* spp. (HS) (WHC: 10.66%; OHC: 13.50%), ratify their physical aptitude for incorporation into industrial pelletizing processes [37] (Figure 8).

### 3.4. Zootechnical Performance and Final Product Quality

While the sociocultural diagnosis prioritized native species, for the experimental protein substitution phase, standardized biological models (*T. molitor* and *A. domesticus*) were selected due to their stable commercial availability and homologizable nutritional profile.

**Figure 9:**
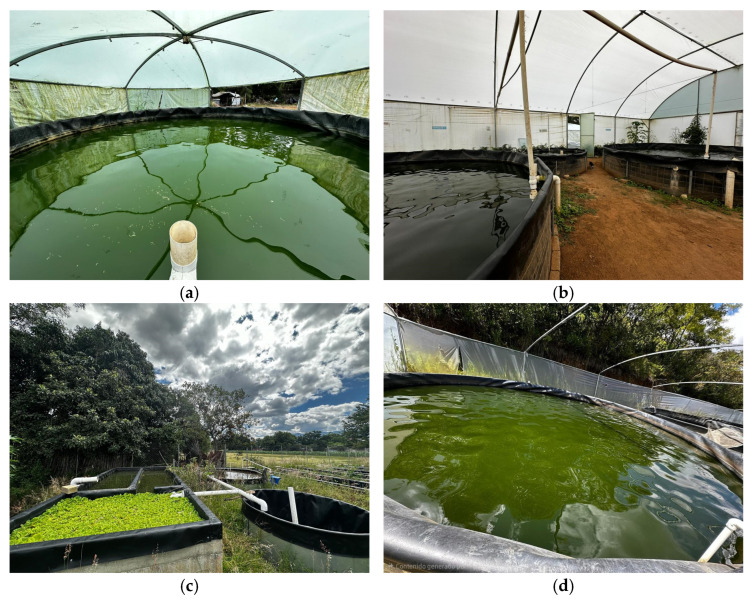
Tilapia aquaculture farms in the Central Valleys of Oaxaca. (**a**,**b**) Under cover and (**c**,**d**) open-air.

The biological efficiency of these flours gains strategic relevance when contrasted with the economic reality of aquaculture in Oaxaca, where commercial feed represents between 50% and 70% of operating costs [8,17]. In this context of high financial vulnerability and water scarcity, feeding trial results show that substituting commercial inputs is technically feasible when considering similar experiments [20,68] (Figure 9).

**Figure 10:**
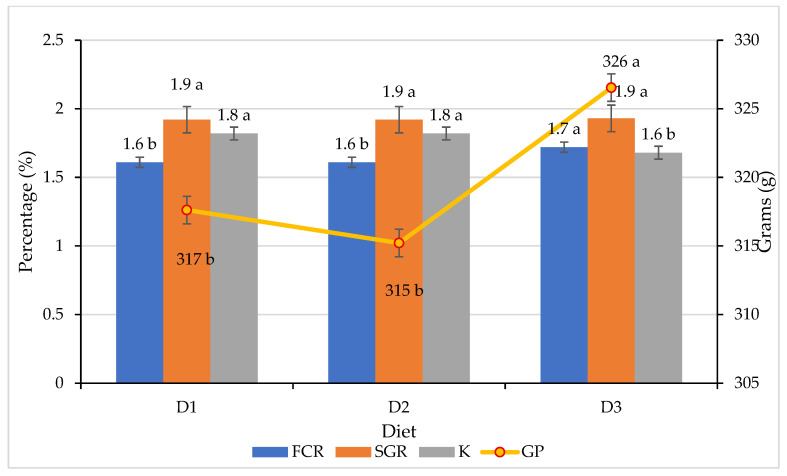
Growth parameters of *Oreochromis niloticus* (Nile tilapia) fed for 280 days. D1 = 100% of *Tenebrio molitor*; D2 = 100% of *Acheta domesticus*; D3 = commercial control; FCR = Feed Conversion Ratio; SGR = Specific Growth Rate; K = Condition Factor and WG = Weight Gain. Data are expressed as mean ± standard error (*n* = 3). Different lowercase letters indicate significant differents between treatments (Tukey’s test, *p* < 0.05). Data adapted from experimental validation.

Productive Viability: Experimental data from the long-term culture (280 days) of *O. niloticus* evidence that total substitution (100%) of commercial feed with insect-based diets (*T. molitor*, D1, and *A. domesticus*, D2) does not compromise growth [15,60]. As shown in Figure 9, statistical analysis revealed significant differences (*p* > 0.05) in Weight Gain (Wg) between the insect-based groups and the commercial control (D3). Specifically, key zootechnical indicators, such as Feed Conversion Ratio (FCR: 1.61–1.62) and Condition Factor (K), were comparable or superior to values reported for the commercial control (D3), with D1 and D2 grouping in the same statistical range as D3 regarding Specific Growth Rate (SGR) (Figure 10).

**Figure 11:**
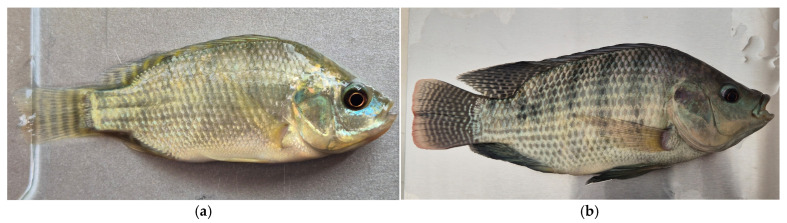
*Oreochromis niloticus* fed with insect-based diets. (**a**) Growth at 40 days and (**b**) Growth at 280 days.

These results validate that it is possible to decouple local production from global inputs without sacrificing performance [20,68] (Figure 11), which aligns with recent literature supporting the inclusion of insect meals. Results regarding the use of *T. molitor* meal have shown adequate digestibility and nutritional utilization in growing and juvenile tilapia, without affecting growth, feed efficiency, or body composition, thus supporting its viability across different stages of the production cycle [47,70].

Similarly, partial substitution (35%) of fishmeal with *A. domesticus* meal did not alter productive performance or survival in juvenile tilapia [71]. Furthermore, in fry, total replacement with *A. domesticus* meal has been reported to improve growth and feed efficiency while maintaining 100% survival [72].

Nutritional Sovereignty: Beyond tank performance, the effect on fillet quality was significant. Proximal analysis of the final product revealed a substantial improvement in the nutritional profile: fish fed with insect-based diets (D1 and D2) presented a statistically higher protein content (11.75% and 11.31%, respectively; *p* < 0.05) compared to the commercial control (8.37%). Furthermore, a total absence of carbohydrates was observed in the insect-fed groups, significantly differing from the control (*p* < 0.05) [1,73,74].

**Figure 12:**
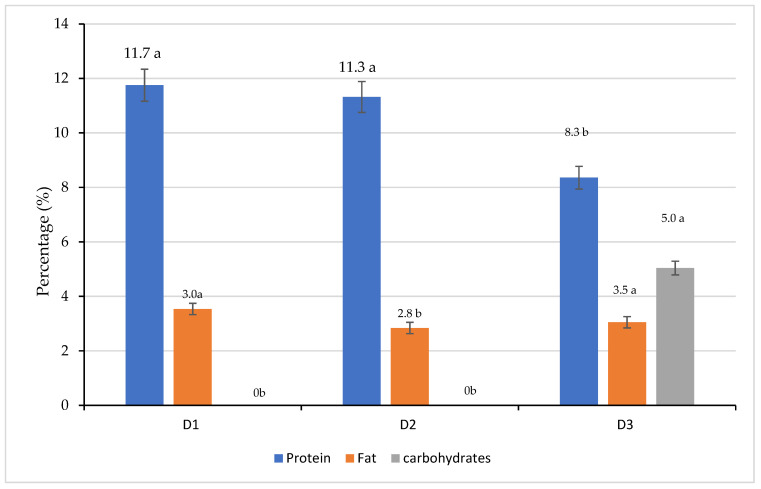
Proximal composition (% dry weight) of fish fillet (*Oreochromis niloticus*) fed with 100% insect flour. D1 = 100% of *Tenebrio molitor*; D2 = 100% of *Acheta domesticus* and D3 = commercial control. Data are expressed as mean ± standard error (*n* = 3). Different lowercase letters indicate significant differents between treatments (Tukey’s test, *p* < 0.05). Data adapted from experimental validation.

This superiority in protein retention is primarily associated with the high chitin and chitosan content in the insect exoskeleton, which acts as a prebiotic and improves nutrient assimilation efficiency in fish [75]. These findings are consistent with studies on rainbow trout (*Oncorhynchus mykiss*) fillets (14–15%) [76] and *O. niloticus* (13–17%) [77] when fed *T. molitor* meal at different concentrations. Regarding carbohydrates, the low levels detected align with the physiological reality of fish muscle, where glycogen content is typically maintained below 0.50% [78]. Consequently, the variations observed in the proximal composition, including lipid content and protein, can be attributed to multifactorial interactions, involving fish age, dietary ingredient quality, and experimental conditions such as temperature and oxygenation [75,76] (Figure 12).

Microbiological Safety: Finally, the microbiological analysis confirmed that the safety guaranteed in the insect flour via thermal processing is effectively transferred to the edible tissue. Pathogens such as total coliforms, fungi, and yeasts were absent in the fillets of all treatment groups, complying with current sanitary safety standards [42,43,44,45,49,79] (Table 6).

Regarding aerobic mesophiles, the counts obtained (5 × 10^4^ − 1 × 10^5^ CFU/g) demonstrate high sanitary quality when compared to the literature. A previous study reported the presence of total coliforms in fresh *Mormyrus kannume*, with Enterobacteriaceae counts ranging between and 1.2 × 10^3^ and 2.7 × 10^3^ CFU/g [80]. Similarly, aerobic mesophilic bacteria were detected in fresh yellow corvina (*Cynoscion xanthulus*) at 3.9 log CFU/g [81]. In *Oreochromis* sp., counts of approximately 6 log CFU/g (1 × 10^6^) of aerobic mesophiles have been documented in semi-intensive systems [82].

**Figure 13:**
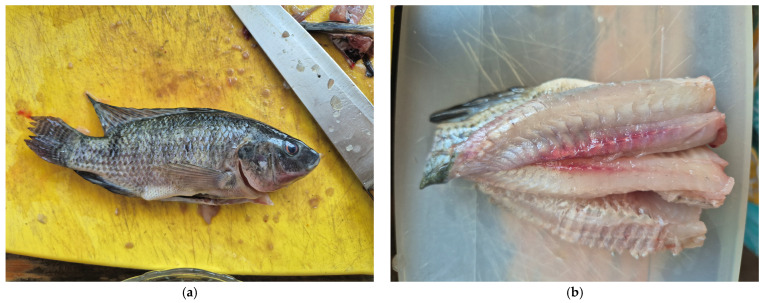
Final product of insect-based diets. (**a**) Whole fish, and (**b**) Safe fillet.

Consequently, the absence of pathogens and the low mesophilic counts in this study empirically validate that utilizing organic waste substrates for insect rearing does not compromise the sanitary quality of the final aquaculture product (Figure 13).

### 3.5. Model Synthesis: Backyard Integrated System

Collectively, scientific evidence supports the use of insect flours as sustainable and efficient alternatives, with the potential to reduce dependence on marine inputs and strengthen the circular economy in aquaculture [47,71,72,83,84]. Hence the integration of these findings—sociocultural/biocultural validation, nutritional safety, and zootechnical efficiency—grounds the design of the Backyard Integrated System. This model is not an isolated theoretical construction, but the functional articulation of previous evidence to solve the dependency on external inputs [1,25,30,47,68,85,86].

Integrated backyard systems combining tilapia production, home gardens, and insect rearing represent a sustainable approach specifically adapted to the socioeconomic requirements of rural communities in Mexico. Previous studies on backyard tilapia production demonstrate that this species can be cultivated viably and self-sufficiently in rural zones, characterized by low implementation and operational costs, thereby contributing to local food security [87]. A pertinent example is found in the low-input backyard cultures of Oaxaca, which typically utilize rustic earth ponds lined with black plastic. In these systems, feeding strategies are often limited to household scraps such as tortilla and dry bread, with production primarily destined for self-consumption or occasional local sale. Another subsistence strategy observed in Oaxacan aquaculture gardens involves the application of mixed systems, where nutrient-rich pond water is repurposed for small-scale vegetable irrigation. This aligns with recent data reporting that nutrient recirculation between tilapia and vegetable crops enhances total system productivity, a critical factor for small-scale producers operating with limited resources [88].

The system aligns with Circular Bioeconomy principles at a family scale, structuring a closed loop that transforms waste into high-value inputs and promotes productive resilience. Crucially, the architecture of this Bio-circular Model is specifically designed for rural contexts characterized by limited capitalization. It operates under a logic of “self-generation”, utilizing low-impact organic waste, resources of negligible commercial cost, to substitute expensive inputs. This approach establishes an accessible technological alternative for communities, reducing their vulnerability to external market volatility.

The operability of this proposal is based on the dynamic interconnection of three subsystems allowing for the closing of technical and biological cycles. The flow begins with the management of Circular Inputs (Module 1), where organic household waste and garden by-products are revalorized as substrate. This strategy is supported by experimental evidence demonstrating the viability of organic residues as feed for edible insects [10]. Specifically, *Tenebrio molitor* can develop effectively on diets based on agro-industrial by-products—such as distillery grains, bread waste, and vegetable remains—without compromising survival or growth rates [11,12,13]. Similarly, for *Acheta domesticus*, it has been reported that diets formulated with mixed food waste and agricultural by-products allow for adequate growth and feed conversion efficiency [14].

Subsequently, these substrates feed the Biological Conversion Module (Module 2), in which controlled insect rearing transforms residual biomass into high-quality protein. This controlled production stabilizes the resource supply, effectively overcoming the limitations of wild seasonality. Beyond protein, this module generates a valuable by-product: insect residues (frass). Literature indicates that frass serves as an effective organic fertilizer for crops, contributing to sustainable agriculture; this aligns directly with the operational logic of backyard circular systems [89]. Thus, insect rearing provides a dual benefit: a high-nutritional-value protein alternative for human and animal consumption and a functional input for agriculture, delivering significant socioeconomic and ecological benefits [89].

**Figure 14:**
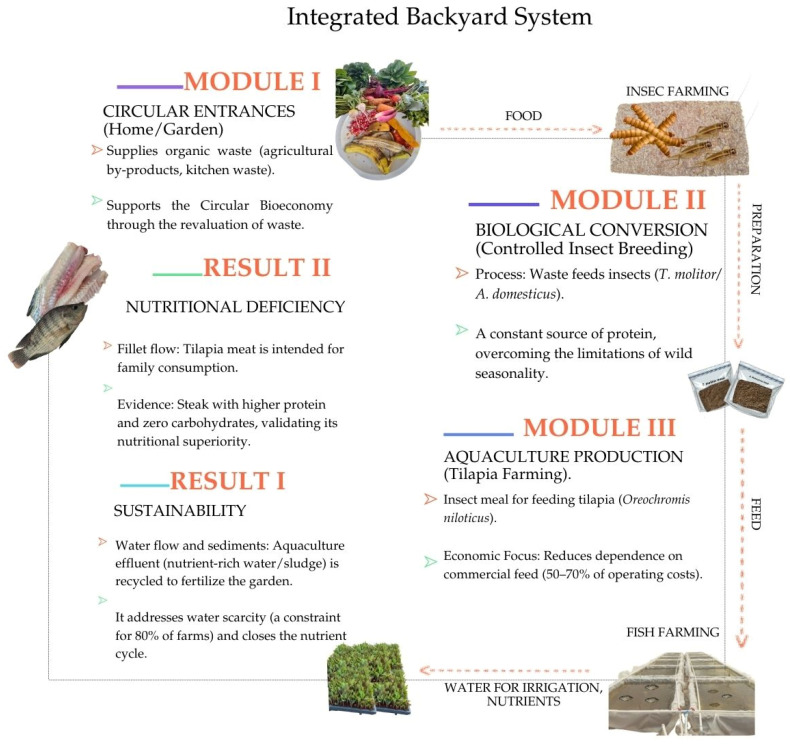
Functional structure of the Backyard Integrated System Model.

Finally, the protein output supports the Aquaculture Production Module (Module 3), allowing for tilapia fattening with reduced dependency on commercial feeds. The system closes the cycle by returning value to the start of the chain: aquaculture effluents, rich in nitrogen and phosphorus, are reused for garden fertigation (Result 1: Sustainability), while the final harvest of fish and vegetables guarantees the availability of safe food for self-consumption (Result 2: Nutritional Sovereignty). Regarding social and economic impact, these integrated backyard systems strengthen household food security and generate supplementary income by enabling families to produce high-nutritional-value foods, specifically tilapia protein and fresh vegetables, with low technological and economic entry barriers. Bioeconomic modeling of backyard tilapia production indicates that these systems can effectively generate surpluses for both self-consumption and local sale; however, profitability remains contingent upon variables such as self-consumption rates and market price fluctuations (Figure 14).

## 4. Conclusions

The research validates that entomophagy in Oaxaca transcends its conception as static cultural heritage to position itself as a dynamic biotechnological solution capable of strengthening regional food sovereignty. It is confirmed that biocultural synergy acts as a catalyst for innovation, where the deep-rooted identity associated with insects mitigates neophobia barriers and facilitates the transition from wild gathering toward controlled production systems.

From a technical standpoint, the study empirically demonstrates that the “Backyard Integrated System” effectively closes the safety and production loops. The absence of pathogens in the final fillet confirms that thermal standardization of insect flours is a sufficient mechanism to guarantee food safety, even when using organic waste as a rearing substrate. Furthermore, the model overcomes the structural barrier of seasonality: while wild harvesting cannot guarantee the continuous supply required for industry, the controlled rearing modules stabilize production year-round.

Economically, the results validate that total substitution of commercial feed with insect biomass is zootechnically feasible. This finding is transformative for rural economies: by utilizing self-generated organic waste as a zero-cost substrate, the model decouples small-scale producers from volatile external markets. This reduction in operational costs (where feed typically represents 50–70% of expenses) establishes a scalable and resilient Circular Bioeconomy framework suited for communities with limited access to capital.

However, a limitation of this study lies in the use of standardized model species (*T. molitor* and *A. domesticus*) for the experimental phase to ensure data consistency, rather than the native species (*Sphenarium* spp. and *Edessa* spp.) that grounded the sociocultural analysis. Consequently, future research perspectives must focus on establishing specific domestication and mass-rearing protocols for these local taxa. Achieving this step will provide the full functional architecture to position native entomophagy not only as heritage but as a scalable biotechnology for future food security.

## Figures and Tables

**Table 1:** Comparative economic baseline: Estimated production costs of insect flours (small-scale) versus commercial aquaculture feed.

Parameter	*Tenebrio molitor*	*Acheta domesticus*
Biological Conversion		
Required Fresh Biomass (to make 1 kg dry)	3.12	3.70
Drying yield (%)	32	27
Estimated Unit Cost		
Production costs (USD/Kg dry) ^1^	6.92	17.30
Commercial Reference (Control) ^2^		
NutriPec^®^ (USD/25 Kg bag)	78.37	78.37
NutriPec^®^ Market Price (USD/kg)	3.13	3.13

^1^ Production costs for insects are estimated based on small-scale operations (subsistence farming). ^2^ Commercial feed prices reflect the average retail cost of NutriPec^®^ (Agribans-Purina, Guanajuato, Mexico) in the Oaxaca region.

**Table 2:** Proximal composition (%) of insect-based diets for tilapia.

Diets	Protein	Lipids	Carbohydrates	Fiber
*T. molitor*	47	33	10.3	6
*A. domesticus*	58	16	25	19
Commercial feed	45	27	20	3

**Table 3:** Diversity of culinary preparations and commercial valorization of insects in Oaxaca.

Culinary Category	Preparation and Context	Insect Input
Typical Dish (Whole)	*Quesillo* empanadas with “chapulines”, quesadillas, omelets, “tasajo” with egg, snacks with or without spicy chili [30].	“Chapulín” (*Sphenarium* spp.)
Typical Dish (Condiment)	Sauces (salsas), moles, chili powder, vinaigrettes, and salt [30,60].	“Chapulín” (*Sphenarium* spp.), “Chicatana” (*A. mexicana*), and “Gusano de Maguey” (*C. redtenbacheri*)
Regional Typical Dish	Sauces and fried snacks (Mixteca Region) [29]	“Cocopaches” (*Thasus gigas***)**
Traditional Drink	Mezcal (for flavoring), cocktails	“Gusano de Maguey” (*C. redten-bacheri*) and “Grana cochinilla” (*Dactylopius coccus*) for coloring (innovative use)
Desserts/Sweets	Chocolate truffles and sorbets	“Chapulín” (*Sphenarium* spp.), “Chicatana” (*A. mexicana*)

**Table 4:** Seasonality, cost, and collection region of edible insects in Oaxaca.

Insect	Place of Purchase	Approximate Cost (MXN)	Consumption Prevalence	Region
“Chapulín” (*Sphenarium* spp.)	Markets, tianguis.	100 g for $1 USD or 1 kg for $27–44 USD	1 and 4 times a month	Central Valleys
“Chicharra” (*Quesada* sp.)	Local markets	100 g for $2 USD	1 and 2 times per season	Central Valleys and Mixtec
“Chicatana” (*Atta mexicana*)	Local markets	In season, 1 kilo for $88 USD. Out of season, 20 g for $4 USD.	1 and 2 times per season	Central Valleys and Mixtec
“Chinche de campo” (*Edessa* spp.)	Local markets and street vendors in the region	10 g for $1 USD	1 and 4 times per season	Mixtec
“Gusano de maguey” (*C. redtenbacheri*)	Markets and mezcal producers	5 g for $13 USD	1 and 2 times per season	Central Valleys

**Table 5:** Seasonality of edible insect collection in Oaxaca.

Insect	Season	Habitat/Collection Sites	Method	Cultural Importance
“Chapulín” (*Sphenarium* spp.)	August–November	Fallow lands, grasslands, and agricultural crops (alfalfa, corn, squash, beans).	Manual harvesting using hand nets, buckets, and plastic bags.	High gastronomic value: consumed habitually by locals and widely sought by tourists.
“Chicharra” (*Q. gigas*)	March–May	Primarily on branches or trunks of “Guamuchil” (*Pithecellobium dulce*) and “Guaje” (*Leucaena* sp.) trees.	Selective trapping using a long reed pole (*Phragmites australis*) tipped with a cut plastic bottle or adhesive tape to capture the insect from high branches.	Traditional local consumption.
“Chicatana” (*A. mexicanq*)	June–July	Open fields, riverbanks, and urban areas (near white light sources).	Manual capture during nuptial flights; use of plastic bags over nests or light traps to attract queens.	High symbolic and gastronomic value; considered a seasonal prestige food.
“Chinche de campo” (*Edessa* spp.)	November–February	Pine-oak forests.	Foraging between soil and leaf litter, or direct collection from tree branches.	Traditional food is strongly associated with local Mixtec identity.
“Gusano rojo de maguey” (*C. redtenbacheri*)	July–October	*Agave* spp. plantations.	Manual extraction as larvae emerges from the leaf, or by uprooting the infested plant to collect from the crown base.	High gastronomic value; key ingredient in traditional Mezcal cuisine and festivities.
“Gusano de picudo del maguey” (*S. acupunctatus*)	Year-round	*Agave* spp. plantations.	Opportunistic harvesting from damaged agaves, specifically during the harvest of agave hearts (piñas) or prior to baking, when larvae are exposed.	Traditional food.
Wasps (Vespidae, subfamily Polistinae)	June–November	Deciduous forests, pine-oak forests, and scrublands.	Nidary extraction by cutting combs from scrub vegetation with a machete. Black combs are typically found higher in trees (e.g., *Prosopis* sp.) than yellow combs.	Traditional food.
“Ticoco” (*M. chevrolatii*)	September–November	Pine-oak forests.	Wood foraging inside decaying logs of oak (*Quercus* sp.), alder (*Alnus acuminata*), white sapote (*Casimiroa edulis*), or *Guaje* (*Leucaena* sp.).	Traditional food.
“Tindaka” (Vespidae, Subfamily Vespinae, *Vespula* sp.)	September–November	Semi-arid soils.	Excavation and smoking; diggers apply heavy smoke to force the queen out before collecting larvae. Process involves high risk of stings.	Traditional food.

**Table 6:** Microbiological quality of *Oreochromis niloticus* fillets fed with insect-based diets compared to commercial feed.

Experimental Diets	Aerobic Mesophiles (CFU/g)	Total Coliforms (CFU/g)	Fungi and Yeasts (CFU/g)	Sanitary Compliance
D1 (*T. molitor*)	1 × 10^5^	<10	<10	Complies
D2 (*A. domesticus*)	3 × 10^4^	<10	<10	Complies
D3 (Control)	5 × 10^4^	<10	<10	Complies
Permissible Limit ^1^	5 × 10^5^	<100	N/A	--

Values expressed as Colony Forming Units per gram (CFU/g). <10 indicates values below the microbiological detection limit (Absence). ^1^ Permissible limits based on NOM-242-SSA1-2009 for fresh fishery products and NOM-093-SSA1-1994.

## Data Availability

The data presented in this study are available on request from the corresponding author. Nutritional characterization data used for the model design are available in previously published works [8,17,28,37] cited in the References.

## Abbreviations

The following abbreviations are used in this manuscript:

FCR	Feed Conversion Ratio
Wg	Weight Gain
K	Condition Factor
SR	Survival
ANOVA	Analysis of Variance
UNESCO	United Nations Educational, Scientific and Cultural Organization
